# Supplementary material for: Association between oral microbiome and seven types of cancers in East Asian population: a two-sample Mendelian randomization analysis
Source: Front Mol Biosci. 2023 Nov 21;10:1327893. doi: 10.3389/fmolb.2023.1327893 (PMC10702768; doi:10.3389/fmolb.2023.1327893)
Supplement: Supplementary file 1 [file DataSheet1.ZIP › Supplementary Materials/MR plots for tongue/tongue═╝/Gastric cancer/pheno.1860_to_gastric cancer_leave_one_out.pdf]

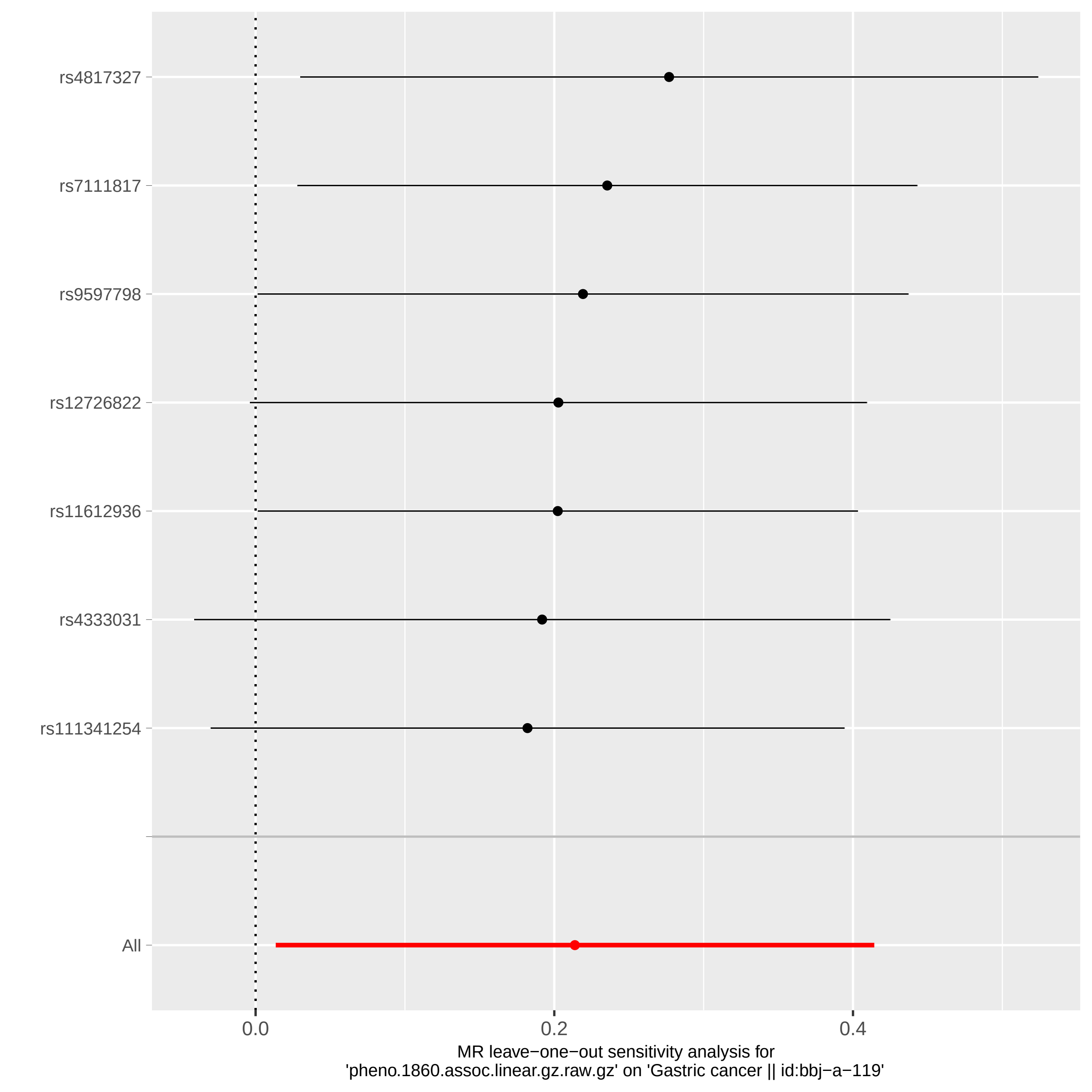
rs4817327
rs7111817
rs9597798
rs12726822
rs11612936
rs4333031
rs111341254
All
0.0
0.2
0.4
MR leave−one−out sensitivity analysis for
'pheno.1860.assoc.linear.gz.raw.gz' on 'Gastric cancer || id:bbj−a−119'
